# Cost of Illness, Quality of Life, and Work Productivity in Axial Spondyloarthritis Patients Receiving Biological Treatments in Greece

**DOI:** 10.31138/mjr.34.1.37

**Published:** 2023-03-31

**Authors:** Nikolaos Kougkas, Charalambos Mylonas, Nestor Avgoustidis, Irini Flouri, Prodromos Sidiropoulos, Theodoros Dimitroulas, Alexandros Garyfallos

**Affiliations:** 1 Fourth Department of Internal Medicine, Hippokration University Hospital, Medical School, Aristotle University of Thessaloniki, Thessaloniki, Greece,; 2 National School of Public Health, Athens, Greece,; 3 Department of Rheumatology, Clinical Immunology and Allergy, University Hospital of Heraklion, Heraklion, Greece

**Keywords:** cost of illness, quality of life, work productivity, axial spondyloarthritis, biological treatments in Greece

## Abstract

**Objectives:**

To estimate the cost of illness, quality of life and work productivity in patients with Axial Spondyloarthritis (Axial SpA) under biological treatment in Greece.

**Methods:**

We conducted a prospective study of 12-month duration, of patients with Axial SpA from a tertiary hospital in Greece. Adult patients fulfilling the Assessment of SpondyloArthritis international Society (ASAS) criteria were enrolled at the beginning of biological treatment due to active disease [Bath Ankylosing Spondylitis Disease Activity Index (BASDAI) >4] and failure of first line treatment. All participants completed questionnaires about quality of life, financial costs and work productivity at the same time with the disease activity assessment.

**Results:**

74 patients of whom 57 (77%) with a paid job, were included in the study. The total annual cost for Axial SpA patients is € 9,012.40 while the average cost of acquisition and administration of the drugs is € 8,364. The mean BASDAI in the 52 weeks of follow-up, was decreased from 5.74 to 3.2 and the mean Health Assessment Questionnaire (HAQ) also from 1.13 to 0.75. Work productivity of these patients as measured with the Work Productivity and Activity Impairment Questionnaire (WPAI), was significantly impaired at the baseline and improved after the initiation of biological treatment.

**Conclusions:**

The cost of illness in patients receiving biological treatments in Greece is high. However, these treatments except from the well-established positive effect on disease activity, can improve remarkably the work productivity and quality of life of Axial SpA patients.

## INTRODUCTION

The concept of Axial Spa was realized in 2009 by ASAS.^1^ It was a result of the need to early recognize and diagnose patients with Ankylosing Spondylitis (AS), which were until then diagnosed according to imaging and clinical criteria of 1984 New York.^2^ The use of MRI and the understanding of the role of HLA-B27 contributed to the identification of patients with all the clinical features of AS but without definite radiographic changes. This subgroup of patients was labelled as non-radiographic Axial Spa in contrast to AS which has the characteristic radiographic changes according to New York criteria. Both entities, AS and non-radiographic Axial Spa, are included in Axial Spa, which is a part of a larger family of diseases, the Spondyloarthropathies.^3^ As the disease starts usually between the second and third decade of life and has a chronic progression, the impact on quality of life, the ability to perform paid work, and health resources is significant.^4^ From a socio-economic point of view, it is important to use therapies that prevent or slow down the progression of the disease, thus avoiding the high economic costs associated with it, the reduction of productivity and the deterioration of the quality of life. The introduction of biological therapies in the treatment of inflammatory diseases has brought impressive results in the course of the disease.^5^ However, their increased financial costs compared to conventional treatments question the use of these drugs and therefore pharmacoeconomic studies are needed to prove the effectiveness of such interventions.^6^ In this study, we aim to estimate the cost of illness, quality of life and workability among patients with axial Spa treated with biologic agents in Greece.

## MATERIALS AND METHODS

The present analysis is a prospective, real, observational, cost-of-illness, and quality of life study. Patients that were followed at the outpatient clinic of the University Hospital of Heraklion, were eligible if they fulfilled the ASAS criteria for Axial SpA and were starting treatment with a biologic agent.

All patients were monitored for 12 months in order to collect strong and representative data. During the follow-up period, two visits were made (one in the middle and another at the end of the 12-month period). If patients were unable to follow up on follow-up visits, they contacted the researcher by telephone to complete the questionnaire.

Data collection was performed through two questionnaires/reference form cases. One was for the patient’s initial visit to the doctor’s office and the second was used for the two follow-up visits which were completed by the same doctor/researcher to collect the required data.

The data collected from the patients was carried out in two stages, with the 1st stage including the collection of quantitative data and the 2^nd^ stage including the collection of qualitative data through an interview guide. The quantitative data, as mentioned, were collected through a personal interview by the researcher himself, after the informed consent of the patients and using the questionnaires HAQ, BASDAI, EuroQol-5 Dimension (EQ-5D), and WPAI. At the same time, various demographics of the participating patients were completed in the questionnaire.

### Baseline clinical and socio-demographic characteristics

For the needs of the present analysis, clinical and socio-demographic data were collected. The participants of the survey initially completed a questionnaire about their social and demographic data. These data were gender, age, marital status, income, work status, and level of education. Regarding the clinical parameters, the patients answered about the disease, its diagnosis, the BASDAI scale, the use of drugs, the Bath Ankylosing Spondylitis Functional Index (BASFI), and other tests such as laboratory and imaging (**Table 1**). All the above characteristics were recorded through questionnaires and face-to-face interviews.

**Table 1.** Baseline clinical and socio-demographic characteristics of AS patients treated with biological agents (n=74).

**Characteristics**	**Mean (SD)**

Age	43.1

Sex (Female)	45 (61%)

Marital status	
Married	57 (77%)
-Single	11(15%)
-Divorced	6 (8%)

Education	
-Primary school	9(12%)
-Secondary school	43(58%)
-College/University	22(30%)

Employment	
Yes	57(77%)
No	13 (17%)
Pension	4(5%)

BASDAI	5,74

BASFI	4,33

EQ-5D	1,74

HAQ	1,13

WPAI	28.57% (3.42%).

### Quality of life instruments

One of the most important parts of the questionnaire was the collection of quality of life (QoL) data. These data were evaluated through WPAI, HAQ, and EUROQOL questionnaires.

WPAI (Work Productivity and Activity Impairment Questionnaire): This questionnaire is a variant of a specific questionnaire developed to measure the impact that a disease has on work productivity. Over the years and its widespread use, this questionnaire has proven its validity, reliability and sufficient predictive value to be able to measure the impact of the disease concerning its absence, presentation, and overall attenuation productivity.^7^ In general, the WPAI questionnaire is a quantitative tool used to measure reduced productivity at work and during leisure activities (ie, typical activities that a person performs regularly, such as housework, shopping, childcare, and exercise). Higher percentage scores are worse in terms of absenteeism, presentation, overall productivity and attenuation of leisure activities.

The HAQ questionnaire was used to monitor the functional capacity of patients with Axial SpA. This questionnaire reflects the impact of the disease on daily life. Contains questions about the ability to perform activities in eight areas (dressing and grooming, climbing stairs, eating, walking, hygiene, approach, grip, and activities). The final score ranges from 0 to 3, where 0 indicates no functional failure and 3 indicates the worst prognosis.^8^

### Cost of Illness (COI)

**Figure 1.**
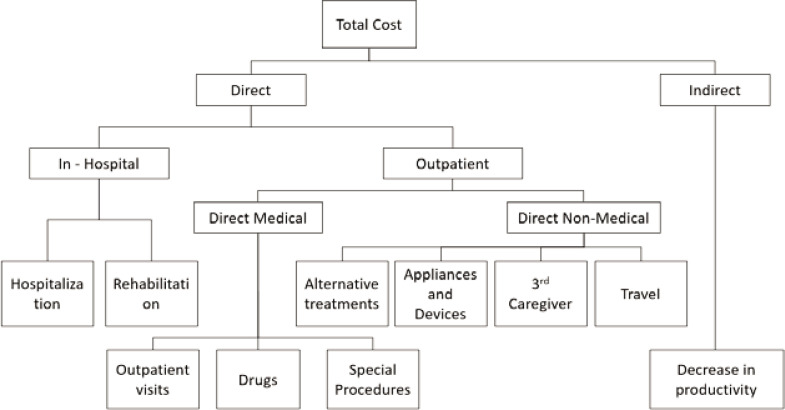
Cost components.

Given that the analysis was carried out by society, all costs (direct and indirect) were considered in the model. The total cost for each health condition reflects and includes all possible health and non-health consumption of patients during treatment (eg, hospitalisation, doctor visits, medications, complementary therapies, laboratory and imaging tests). The use of resources associated with each health condition was based on the experience of both the expert/author and the data collected from the questionnaires completed by the patients themselves (**Figure 1**). The resources consumed were combined with the corresponding costs to estimate the total costs. All costs applicable to the analysis relate to the year 2021.

## RESULTS

### Patient Demographics

The analysis included a total of 74 patients over 18 years old, from February 2016 to July 2019. The majority fulfilled also the modified New York criteria for AS while the rest were classified as Non-Radiographic Axial SpA. The size and distribution of the sample were based on data based on attendance at the hospital clinic and its size was calculated using the power analysis method to include all the necessary reliability criteria. The sample size was estimated to compare the two groups using the G-tool power.

Within a year, all the expected questionnaires were received, and no patient had to be excluded due to any reason (eg, missing data). Demographics of the patients are presented in **Table 1**. The mean age of interviewees was 43.1 years with a mean disease duration of 9.2 years. The mean BASDAI was 5.74 ± 1.06 and the mean BASFI 4.33 ± 0.97. Quality of life (QOL) measured with EuroQol visual Analog Scale and HAQ was 1.74 (SD: 0,68) and 1.13 (SD: 0,11), respectively. The mean (SD) activity impairment of all patients due to illness – health measured with WPAI was 28.57% (3.42%).

**Table 2.** Correlation of productivity gain and decrease of HAQ, BASDAI.

**WPAi**	**HAQ**					
	0 – 0.5	0.6 – 1.0	1.1 – 1.5	1.6 – 2.0	2.0 – 3.0	P - value
	14.4%	38.1%	54.4%	76.6%	100%	<0.001
	BASDAI					
	0 – 1	2 – 5	6 – 8	9 – 10		
	7.3%	36,7%	72.3%	100%		<0.001

BASDAI, Bath Ankylosing Spondylitis Disease Activity Index; HAQ, Health Assessment Questionnaire; WPAI, Work Productivity and Activity Impairment.

Most of the patients were female (61 %) and almost 50% had minimal functional impairment (BASFI < 3). 77% of patients were employed or self-employed, which is very similar to an age-matched population in the general population in Greece (National Statistical Service). Based on the analysis, disease activity was not related to age or disease duration, but despite this, the working population in the sample decreased from 65% at BASDAI < 3 to 49% at BASDAI ≥ 7. Functional disability was correlated with age, which shows that there is a strong effect of functional disability on people’s ability to work. The proportion of workers declined from 78% at BASDAI < 3 to 39% at BASDAI ≥ 7.

#### Quality of Life

##### Improvements in Disease Activity, Quality of Life, and Work Status After Biological Treatments

**Figure 2.**
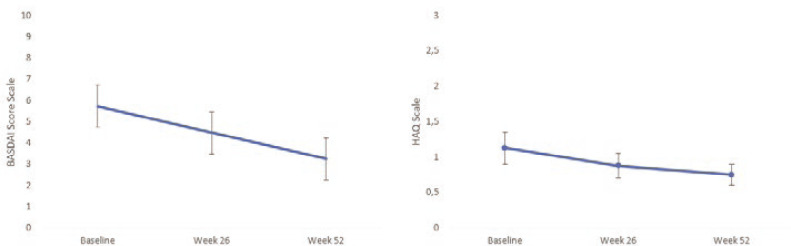
BASDAI (Disease activity) and HAQ (quality of life) change before and after biological treatment. BASDAI: Bath Ankylosing Spondylitis Disease Activity Index; HAQ: Health Assessment Questionnaire

Patients were monitored 3 times a year: at time 0 (baseline) at their initiation of treatment, at 6 months and at 12 months after the initial visit. During these periods, the progression of the disease, as well as their quality of life and productivity were monitored. All patients received biologic therapy at their initial visit. BASDAI and HAQ improvements from baseline to week 26 and 52 were from 5.74 to 3.25 and from 1.13 to 0.75, respectively (**Figure 2**).

**Figure 3.**
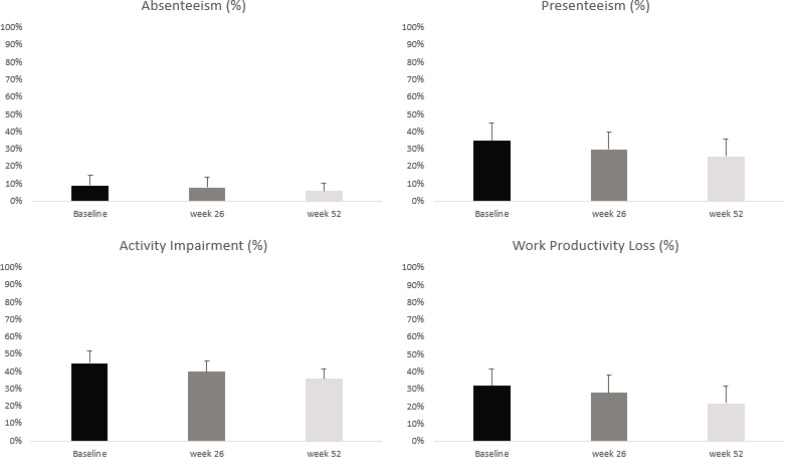
Change in WPAI score scale after biological treatment.

Same results were found in work outcomes including absenteeism, presenteeism and work productivity loss at baseline, week 26 and week 52 (**Figure 3**).

##### Correlation between Quality of Life and Loss of Productivity

In addition, a multi-linear regression model was developed to identify the factors that affect overall productivity loss for patients with Axial SpA surveyed through the questionnaires they completed. The patient’s functional status, as expressed through the HAQ and EuroQol questionnaires is a statistically significant factor (P<0.001) that affects the loss of productivity in patients with Axial SpA. At the same time, it was estimated that if the functional status of patients changes how much it will affect the productivity of patients. More specifically, if the HAQ score decreases by 0.34, the productivity loss will decrease by 5.9%. Whereas, if the BASDAI rating improves by 1 point then the loss of productivity will be reduced by 7.3%. However, the other variables used in the model affected the loss of productivity in Axial SpA patients only numerically without statistical significance.

### Cost of Illness

#### Direct Medical Cost

The direct cost of health care reflects and includes all the consumption of resources incurred for the care of patients in the health care system. Therefore, they include items such as hospitalizations, medications, laboratory, and imaging diagnostic tests. In the present analysis, the drug acquisition cost (biological agents) that resulted from the questionnaires were calculated. Drugs that were not taken by the participants were excluded from the analysis. The total cost of the drug per month was calculated by combining the dosing schedule of the drugs with the corresponding drug prices, obtained from the last price list issued by the Greek Ministry of Health in 2021. The calculation of the cost of drugs was derived from the way in which each drug is provided through the health care system [hospital pharmacies, pharmacies of National Organisation for the Provision of Health Services (EOPYY) and the way of administration of these drugs (intravenous [IV], subcutaneously [SC], or orally). The percentage and the distribution of patients who received each biological drug were obtained from the questionnaires that were completed by the patients. Taking into account the analysis that presents the annual cost of acquisition and administration of each drug as well as the distribution of patients on drugs (based on the data obtained from the questionnaires), the average annual cost of acquisition of drugs is €7,407 and the average annual cost of administration is €957. The total average annual of the acquisition and administration of medicines is €8,364 (**Table 3**).

**Table 3.** Total annual cost per patient.

	**Payer Reimbursement (EOPYY)**		**Patient Co-payment**	
	**Mean**	**Standard Deviation**	**Mean**	**Standard Deviation**
Direct Medical Cost	8,503.79	347,32	24.61	1.12
Drug Acquisition	7,407.00	311,09	0	0
Administration	957.00	28,71	0	0
Laboratories Tests	77.46	4,33	13.61	0.76
Imaging Tests	62.33	3,17	11	0.56
Direct non-medical costs	0.0	0	430,71	41,87
Indirect Cost	0.0	o	484	32.42
**Total Annual Cost per patient**	8,503.79	347,32	939,32	78.75

#### Axial SpA management (Laboratory and Imaging tests)

The cost of managing Axial SpA for patients arose from the total resources spent on laboratory tests and imaging tests during the year. It should be noted that, to avoid invalid assumptions about the proportion of patients treated in the public or private sector, it was considered that all patients were treated in the public sector. The average total annual cost for laboratory tests for the follow-up of patients with AS amounts to €91.07, of which €77.46 is the cost of EOPYY and €13.61 is the co-payment of patients. The average annual cost for the imaging exams amounts to €73.34 (EOPYY: €62.33, Patient co-payment: €11). The total average annual cost for the follow-up of patients with AS amounts to €164.40 (EOPYY: €139.79, Patient co-payment: €24.61) (**Table 3**).

#### Direct Non-Medical Cost

Data were collected on transportation costs representing all patient travel costs to and from hospitals. Patients’ addresses were used to estimate their distance from the hospital. A standard per-kilometre invoice was then applied to calculate their travel expenses for each hospital visit. The total cost of travel during the 1-year follow-up period was calculated by multiplying the cost of travel for each visit by the number of visits for each participant. The study also collected data on other costs incurred by the patient for various reasons related to the disease, such as out-of-pocket payments for appliances and in-home adjustments (**Table 3**).

#### Indirect Cost

Indirect costs reflect the loss of productivity caused by patients’ inability to work as a result of their condition or because relatives must take leave to care for them. The loss of income from ankylosing spondylitis-related morbidity of participants of productive age was conservatively estimated by multiplying the annual number of days of leave due to Axial SpA by their average daily earnings. The cost of a lost working day is calculated as the gross domestic product per capita (€17,497) based on the latest data from the National Statistical Service (www.statistics.gr) divided by 300 (ie, the number of working days per year). Completion of the questionnaires, both at the initial visit and at follow-up visits (at week 26 and 52), showed that on average the participating patients lost 8.3 working days either because they had to manage their illness (eg, had to undergo examinations) or because they could not work. Therefore, the average annual average cost for patients with Ankylosing Spondylitis amounted to €484 per year (**Table 3**).

## DISCUSSION

This is the first time in Greece that a prospective pharmacoeconomic study is carried out on Axial SpA.^9^ The average annual total cost from the payer’s perspective amounts to € 8,504. The largest part of the cost (~87%) is due to the cost of obtaining the biological treatment and then a very significant contribution to the total cost has the administration cost of drugs (11%). From the patient’s perspective, the average annual total cost is €939. Most of the cost (~52%) corresponds to the indirect cost, ie, the loss of productivity caused by the inability of patients to work as a result of their condition or because relatives have to take a leave to care for them. A significant contribution to the total cost has also the direct non-medical costs (46%), which include the costs of transporting patients to and from the hospital, and home adjustments.

Regarding work productivity, an improvement of disease activity as measured with BASDAI is associated with an increase in performance at work.^10^ In our study, the mean BASDAI of the patients starting biological treatment decreased from 5.74 to 3.2 after 52 weeks while work productivity loss decreased from 30% to 20% accordingly. Similarly, absenteeism and presenteeism also decreased from 9% and 32% to 5% and 23%. The same positive effect on the work productivity of patients with AS was documented by improving their quality of life. The data collected from the HAQ questionnaires showed a reduction of the scale from 1.13 at baseline to 0.75 at the end of the follow-up period. The HAQ and BASDAI scores according to our regression analysis were the factors with the most important predictive value for the total loss of productivity and therefore for the costs that accompany it.

This analysis contains some limitations which should be emphasised. it should be noted that the survey did not collect specific data on the indirect non-medical costs associated with transporting patients to attend visits, examinations, emergencies and other health services, but was assessed indirectly (extrapolation methodology used). Similarly, it did not include direct information about the professional and informal care that patients receive, nor about the adjustments made to housing as a result of physical limitations. Therefore, in many cases and where necessary to estimate the costs associated with informal care and housing adjustments, data were obtained from the literature. Also, in Greece, there is no accurate accounting system of costs and compensation for cost estimation on a patient basis. Therefore, the same daily cost for a hospital stay (including hospital bed, nursing services, doctor and therapist time, and impact management) has been used for all participants in the analysis.

In conclusion, there is a significant cost (direct and indirect) related to the treatment of Axial SpA in Greece. Functional impairment is the factor with the most significant cost impact. Biologic therapies that reduce functional impairment can be effective in lowering the cost of the disease, improving the patient’s quality of life, and reducing stress in the health care system.

## ABBREVIATIONS

AS: Ankylosing Spondylitis
ASAS: Assessment of SpondyloArthritis international Society
Axial SpA: Axial Spondyloarthritis
BASDAI: Bath Ankylosing Spondylitis Disease Activity Index
BASFI: Bath Ankylosing Spondylitis Functional Index
EOPYY: National Organization for the Provision of Health Services
EQ-5D: EuroQol- 5 Dimension
HAQ: Health Assessment Questionnaire
WPAI: Work Productivity and Activity Impairment Questionnaire
